# Impact of clinical osteoarthritis of the hip, knee and hand on self-rated health in six European countries: the European Project on OSteoArthritis

**DOI:** 10.1007/s11136-015-1171-8

**Published:** 2015-11-07

**Authors:** N. M. van Schoor, S. Zambon, M. V. Castell, C. Cooper, M. Denkinger, E. M. Dennison, M. H. Edwards, F. Herbolsheimer, S. Maggi, M. Sánchez-Martinez, N. L. Pedersen, R. Peter, L. A. Schaap, J. J. M. Rijnhart, S. van der Pas, D. J. H. Deeg

**Affiliations:** Department of Epidemiology and Biostatistics, EMGO Institute for Health and Care Research, VU University Medical Center Research, Van der Boechorststraat 7, 1081 BT Amsterdam, The Netherlands; Department of Medical and Surgical Sciences, University of Padova, Padua, Italy; National Research Council, Aging Branch, Institute of Neuroscience, Padua, Italy; Unit of Primary Care and Family Medicine, Faculty of Medicine, Universidad Autonoma de Madrid, Madrid, Spain; MRC Lifecourse Epidemiology Unit, Southampton General Hospital, Southampton, UK; Bethesda Geriatric Clinic, University of Ulm, Ulm, Germany; Institute of the History, Philosophy and Ethics of Medicine, University of Ulm, Ulm, Germany; Department of Medical Epidemiology and Biostatistics, Karolinska Institutet, Stockholm, Sweden; Department of Health Sciences, Faculty of Earth and Life Sciences, VU University Amsterdam, Amsterdam, The Netherlands

**Keywords:** Europe, General population, Osteoarthritis, Self-rated health

## Abstract

**Purpose:**

Osteoarthritis (OA) has been shown to be associated with decreased physical function, which may impact upon a person’s self-rated health (SRH). Only a few studies have examined the association between OA and SRH in the general population, but to date none have used a clinical definition of OA. The objectives are: (1) To examine the cross-sectional association between clinical OA and fair-to-poor SRH in the general population; (2) To examine whether this association differs between countries; (3) To examine whether physical function is a mediator in the association between clinical OA and SRH.

**Methods:**

Baseline data of the European Project on OSteoArthritis (EPOSA) were used, which includes pre-harmonized data from six European cohort studies (*n* = 2709). Clinical OA was defined according to the American College of Rheumatology criteria. SRH was assessed using one question: How is your health in general? Physical function was assessed using the Western Ontario and McMaster Universities OA Index and Australian/Canadian OA Hand Index.

**Results:**

The prevalence of fair-to-poor SRH ranged from 19.8 % in the United Kingdom to 63.5 % in Italy. Although country differences in the strength of the associations were observed, clinical OA of the hip, knee and hand were significantly associated with fair-to-poor SRH in five out of six European countries. In most countries and at most sites, the association between clinical OA and fair-to-poor SRH was partly or fully mediated by physical function.

**Conclusions:**

Clinical OA at different sites was related to fair-to-poor SRH in the general population. Most associations were (partly) mediated by physical functioning, indicating that deteriorating physical function in patients with OA should be a point of attention in patient care.

## Introduction

Musculoskeletal conditions, among which osteoarthritis (OA), are the second greatest cause of disability worldwide according to a new global study on the burden of diseases [1]. OA is a disease defined by characteristic structural alterations of the joint, including focal degradation of articular cartilage and remodeling of subchondral bone with the formation of osteophytes at the joint margins, as well as an illness defined by a person’s symptoms, including pain, fatigue, mood alterations and sleep disturbance [2, 3]. The prevalence of osteoarthritis varies widely between studies and depends on study population, site of interest and definition used [4]. The knee, hip and hand are most affected by the disease [4]. The prevalence of OA increases with age with osteoarthritic changes uncommon under the age of 40, but seen in most people over the age of 70 [5]. In a previous publication from the European Project on OsteoArthritis (EPOSA), 6.1 % of the subjects aged 65–80 years had clinical OA of the hip, 20.2 % had clinical OA of the knee, and 17.1 % had clinical OA of the hand [6].

Self-rated health (SRH) gives an individual’s perspective of his or her overall health and is an important predictor of future health outcomes, such as health service use and mortality [7–9]. In addition, SRH predicts social, mental and physical health outcomes after total joint replacement [10], and in individuals who have symptomatic knee OA [11]. From the latter two studies [10, 11], it was concluded that SRH could be a simple and efficient tool to inform the clinician about multiple health outcomes in patients having OA. Whether OA itself impacts SRH is less clear. This should preferably be examined in the general population, in which the complete spectrum of mild to severe OA is included, as well as individuals without OA.

Few studies have examined the association between OA and SRH in the general population and most of these studies observed an association between OA and poor SRH [12–18]. In these studies, different definitions of OA were used, varying from pain in the hip or knee [13] to a self-reported diagnosis, i.e., self-reported medical provider diagnosis of OA [14, 15] and self-reported arthritis (or rheumatism) [12, 16–18]. To our knowledge, no studies have been performed using a clinical definition of OA in the general population. Furthermore, all of these studies were performed in one country only. Based on differences in the prevalence of clinical OA [6], and differences in SRH [19, 20] between countries, it can well be expected that the association between OA and SRH differs between countries. In other words, country could be an effect modifier in the association between OA and SRH.

Furthermore, earlier studies have shown that OA is associated with functional limitations [21], and functional limitations have been associated with fair-to-poor SRH [20, 22]. Therefore, it was hypothesized that poor physical functioning may be an important mediator in the association between clinical OA and SRH.

The first objective of the current study is to examine the cross-sectional association between clinical OA of the hip, knee and hand and self-rated health in the general population. The second objective is to examine whether this association differs between countries, i.e., whether country is an effect modifier. The third objective is to examine whether the association of clinical OA with SRH is mediated by physical function.

## Materials and methods

### Design and participants

Baseline data from the EPOSA study are used, which includes preharmonized data from six European countries, i.e. Germany (GER), Italy (IT), the Netherlands (NL), Spain (ES), Sweden (SWE), and the United Kingdom (UK). The EPOSA study focuses on the personal and societal burden of OA, and its determinants, in older persons. A detailed description of the study design and data collection of the EPOSA study is described elsewhere [6]. In summary, random samples were taken from five existing population-based cohorts (GER, NL, ES, SWE, UK). In IT, a new sample was drawn. A total of 2942 respondents (response rate, ranging from 64.6 to 82.2 %) was included. The age-range was between 65 and 85 years in most countries except for the UK, which had an age-range of 71–79 years. For the current study, all participants having data on clinical OA, SRH and covariables were included (*n* = 2709). Non-response analyses showed that persons not included in the current study (*n* = 233) were significantly older (75.3 vs 74.1 years, *p* = 0.003) and had lower education (17.0 vs 10.8 % elementary school not completed, *p* = 0.025) as compared with persons included in the current study (*n* = 2709). No statistically significant differences were observed with regard to gender or number of chronic diseases (data not shown). Participants were interviewed by a trained researcher at home or in a clinical center, using a standardized questionnaire and a clinical exam. For all six countries, the study design and procedures were approved by the Medical Ethics committee of the respective centers.

### Clinical OA

Algorithms for clinical OA of the hip, knee and hand were developed based on the American College of Rheumatology (ACR) classification criteria [23] and were based on both self-report and physical examination. The diagnosis of clinical hip OA was present in case of: pain in the hip as evaluated by the Western Ontario and McMaster Universities OA Index (WOMAC) pain subscale score, plus all of: pain associated with hip internal rotation in at least one side; morning stiffness lasting <60 min as evaluated by the WOMAC stiffness subscale; and over 50 years of age [24, 25]. The diagnosis of clinical knee OA was present in case of: pain in the knee as evaluated by the WOMAC pain subscale score, plus any 3 of: over 50 years of age; morning stiffness lasting <30 min as evaluated by the WOMAC stiffness subscale; crepitus on active motion in at least one side; bony tenderness in at least one side; bony enlargement in at least one side; no palpable warmth of synovium in both knees. The diagnosis of clinical hand OA was present in case of: pain, aching or stiffness of the hand as evaluated by the Australian/Canadian OA Hand Index (AUSCAN) pain and stiffness subscale [26, 27], plus any 2 of: hard tissue enlargement of 2 or more of the 2nd and 3rd distal interphalangeal (DIPs), 2nd and 3rd proximal interphalangeal (PIPs), 1st carpometacarpal (CMC) joints of at least one hand; hard tissue enlargement of 2 or more DIPs of at least one hand; deformity of at least 1 of the 2nd and 3rd DIPs, 2nd and 3rd PIPs, 1st CMC joints of at least one hand. Swelling of the metacarpophalangeal (MCP) joints, which is also included in the ACR classification criteria as a control to exclude rheumatic arthritis, was only measured in the UK and Germany.

### Self-rated health

Self-rated health was assessed by the following question “How is your health in general?” Response categories were: very good, good, fair, poor, very poor [28, 29]. Because of the relatively low prevalence of clinical OA in a population-based sample, and a relatively low number of persons having very poor or poor SRH, the outcome self-rated health was dichotomized into fair-to-poor SRH (answer categories “fair” to “very poor”) versus good SRH (“good” to “very good”). This dichotomization has been applied in previous studies [30].

### Potential confounders

Potential confounders were: age, sex, educational level, and number of chronic diseases. Age and sex were already available in the individual cohorts. Educational level was assessed by asking for the highest level of education completed, i.e., elementary school not completed, elementary school completed, vocational education/general secondary education, and college or university education. Number of chronic diseases was assessed by asking for the presence of the following chronic diseases: chronic non-specific lung disease, cardiovascular disease, peripheral artery disease, stroke, diabetes, cancer, and osteoporosis. These chronic diseases were selected based on prevalence and functional consequences. The number of chronic diseases was categorized into 0, 1, 2 or more chronic diseases.

### Potential mediator

A potential mediator was physical function. Physical functioning was assessed by the physical function subscales of the WOMAC and AUSCAN. The WOMAC physical function subscale contains seventeen items concerning the degree of difficulty with knee and/or hip function experienced in the previous 48 h. The AUSCAN physical function subscale contains nine items concerning the degree of difficulty with hand function experienced in the previous 48 h. The WOMAC and AUSCAN responses were scaled on a five-point Likert scale ranging from none (0) to extreme difficulty (4). For both the WOMAC and AUSCAN, missing values were imputed according to the user manual, and subscale scores were normalized resulting in subscale scores ranging from 0 (no difficulties) to 100 (extreme difficulties) [24, 26].

### Statistical analyses

First, differences were tested between persons having fair-to-poor SRH versus good SRH, between persons having clinical OA versus no clinical OA, and between non-responders versus responders. Differences in mean were tested using *T* test for normally distributed variables, differences in median were tested using Mann–Whitney *U* Test for skewed variables, and differences in frequencies were tested using Pearson Chi-square test. Furthermore, differences between countries were tested using Anova for normally distributed variables, Kruskal–Wallis *H* test for skewed variables, and Pearson Chi-square test for frequencies. In the above analyses, sample weights were used to adjust for differences in age and sex distribution across country samples. Logistic regression analyses were used to analyze the association between clinical OA and fair-to-poor SRH. As a first step, it was tested whether country was an effect modifier in a model including age, sex and country by adding the interaction term clinical OA*country. In these analyses, country was analyzed in dummies with the UK as reference group. In case of an interaction effect (*p* < 0.10), the method of Figueiras was applied to obtain country-specific associations [31] and additional confounders (educational level and number of chronic diseases) were added to the model. The above analyses were performed in IBM SPSS Statistics version 22.

Second, it was tested whether physical function was a mediator by decomposing the total effects of OA on SRH into direct and indirect effects. A sequence of multivariable standardized logistic regression analyses was used to calculate the total, direct and indirect effects for each country. All analyses were adjusted for confounding variables. The total effect is the effect of OA on fair-to-poor SRH. The direct effect is the effect of OA on fair-to-poor SRH after adjustment for physical function. The indirect effect is the multiplication of the effect of OA on physical function and the effect of physical function on fair-to-poor SRH after adjustment for OA [32]. In other words, the indirect effect quantifies the effect of OA on SHR that is mediated/channeled through physical function.

Because the high number of persons scoring 0 on the WOMAC and AUSCAN physical function domains, and the highly skewed distribution of these variables, these variables were dichotomized: quartile 4 (people having most difficulties) versus quartiles 1–3. This dichotomization has been applied before (e.g. [33]). Regression coefficients were standardized prior to multiplication to make the scales of the two regression coefficients comparable [34]. Since the indirect effect usually has a skewed distribution, bootstrapping using 5000 replications was used to calculate the 95 % confidence interval [35]. Mediation analyses were performed in R statistical software version 3.1.1. To calculate the bootstrap confidence intervals, the package ‘boot’ was used [36].

## Results

In total, 161 persons (5.9 %) had clinical hip OA, 532 persons (19.6 %) had clinical knee OA and 440 persons (16.2 %) had clinical hand OA. The prevalence of clinical OA in each country separately was presented elsewhere [6].

**Fig. 1:**
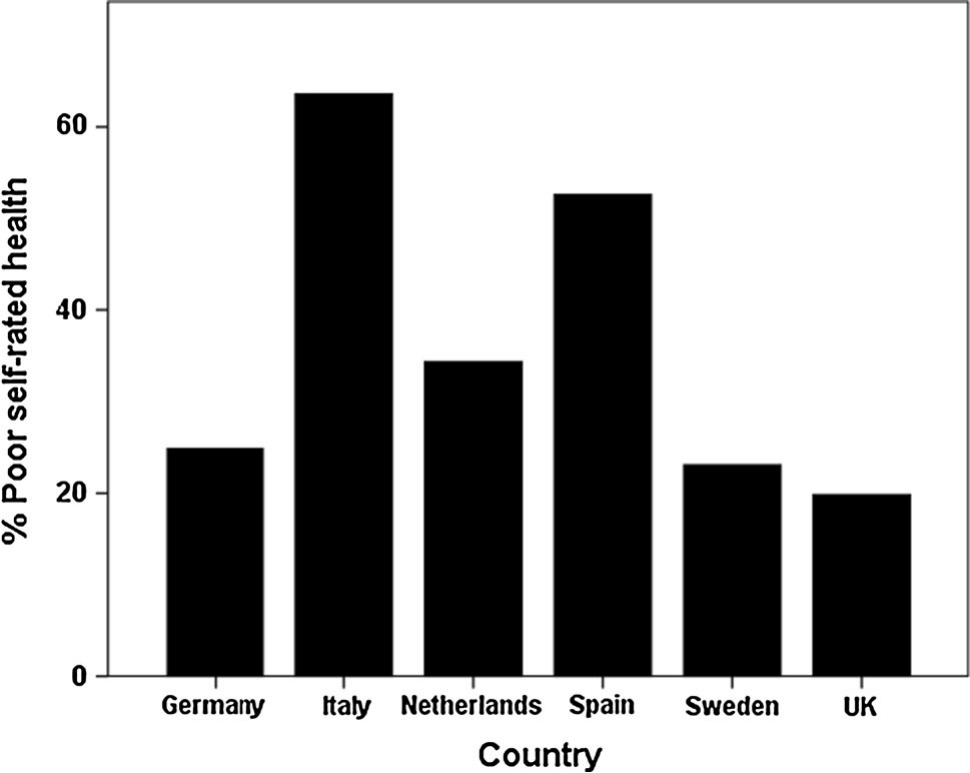
Country differences in fair-to-poor SRH

In Tables 1, 2 and 3, the characteristics of the study sample are described. Persons with fair-to-poor SRH were significantly older, more often female, had a lower educational level, more chronic diseases and higher scores on the WOMAC and AUSCAN physical function domains as compared with persons reporting good SRH (*p* < 0.001 for all variables). Furthermore, persons with clinical knee OA were significantly older than persons without clinical knee OA (*p* = 0.005) and more women had clinical OA of the hip, knee and hand (*p* < 0.001 for all sites). Persons with clinical OA had a lower educational level (*p* < 0.001 for hip and knee OA; *p* = 0.003 for hand OA), more chronic diseases (*p* < 0.001 for all sites), and higher scores on the WOMAC and AUSCAN physical function domains (*p* < 0.001 for all sites). Furthermore, country differences were observed for all variables (Table 3). In Fig. 1, country differences in the frequency of fair-to-poor SRH are presented. It can be seen that the percentage of persons rating their health as fair-to-poor was especially high in IT and ES.

**Table 1:** Baseline characteristics, weighted, according to SRH (*n* = 2709)

	Fair-to-poor SRH	Good SRH	*P* value
Age^a^	74.7 (5.9)	73.2 (5.3)	<0.001
Sex (female)^b^	63.3	52.7	<0.001
Educational level^b^			
Elementary school not completed	16.8	7.8	<0.001
Elementary school completed	45.6	28.5	
Vocational education or general secondary education	25.8	37.2	
College or university education	11.8	26.5	
No. of chronic diseases^b^			
0	15.7	39.5	<0.001
1	35.1	37.6	
2 or more	49.1	22.9	
WOMAC Physical function score hip^c^	1.5 (0–16.2)	0 (0–0)	<0.001
WOMAC Physical function score knee^c^	4.4 (0–19.1)	0 (0–2.9)	<0.001
AUSCAN Physical function score^c^	3.1 (0–19.4)	0 (0–5.6)	<0.001

Differences in mean were tested using *T* test for normally distributed variables, differences in median were tested using Mann–Whitney *U* Test, and differences in frequencies were tested using Pearson Chi-square test

*SRH* self-rated health, *WOMAC* Western Ontario and McMaster Universities OA Index, *AUSCAN* Australian/Canadian OA Hand Index

^a^Mean (SD); ^b^ percentage; ^c ^median (IQ range)

**Table 2:** Baseline characteristics, weighted, according to clinical OA of the hip, knee and hand (*n* = 2709)

	Hip OA	No hip OA	*P* value	Knee OA	No knee OA	*P* value	Hand OA	No hand OA	*P* value
Age^a^	73.2 (5.4)	73.8 (5.6)	0.227	74.4 (5.7)	73.6 (5.6)	0.005	73.5 (5.4)	73.8 (5.6)	0.424
Sex (female)^b^	73.1	55.6	<0.001	70.9	53.1	<0.001	78.8	52.3	<0.001
Educational level^b^									
Elementary school not completed	14.5	10.9	<0.001	18.2	9.4	<0.001	14.7	10.5	0.003
Elementary school completed	48.4	34.0		40.2	33.5		39.1	34.0	
Vocational education or general secondary education	25.2	33.5		28.5	34.1		28.5	33.8	
College or university education	11.9	21.6		13.2	23.0		17.7	21.7	
No. of chronic diseases^b^									
0	16.9	31.5	<0.001	20.9	33.0	<0.001	24.2	31.8	<0.001
1	36.2	36.7		33.7	37.4		34.6	37.1	
2 or more	46.9	31.8		45.4	29.6		41.2	31.0	
WOMAC Physical function score^c^	26.5 (13.9–43.5)	0 (0–1.6)	<0.001	19.3 (8.8–33.8)	0 (0–2.3)	<0.001	–	–	–
AUSCAN Physical function score^c^	–	–	–	–	–	–	22.2 (8.3–41.7)	0 (0–5.6)	<0.001

Differences in mean were tested using *T* test for normally distributed variables, differences in median were tested using Mann–Whitney *U* Test for skewed variables, and differences in frequencies were tested using Pearson Chi-square test

*OA* osteoarthritis, *WOMAC* Western Ontario and McMaster Universities OA Index, *AUSCAN* Australian/Canadian OA Hand Index

^a^Mean (SD); ^b^ percentage; ^c^ median (IQ range)

**Table 3:** Baseline characteristics, weighted, according to country (*n* = 2709)

	GER	IT	NL	ES	SWE	UK	*P* value
Age^a^	74.0 (5.4)	72.6 (5.5)	74.7 (6.2)	74.5 (6.0)	71.6 (5.4)	75.3 (2.6)	<0.001
Sex (female)^b^	47.3	57.0	58.2	55.5	64.1	55.8	<0.001
Educational level^b^							
Elementary school not completed	2.3	7.4	5.9	33.7	11.5	0	<0.001
Elementary school completed	48.2	70.2	18.5	37.8	14.4	20.2	
Vocational education or general secondary education	29.3	21.6	55.6	15.4	31.6	45.4	
College or university education	20.3	0.9	19.9	13.0	42.4	34.4	
No. of chronic diseases^b^							
0	23.9	19.0	33.1	19.9	47.6	42.4	<0.001
1	36.5	42.0	34.7	35.6	34.5	37.1	
2 or more	39.6	38.9	32.1	44.5	17.8	20.5	
WOMAC physical function score hip^c^	0 (0–0)	0 (0–10.0)	0 (0–5.9)	0 (0–5.0)	0 (0–5.0)	0 (0–0)	<0.001
WOMAC physical function score knee^c^	0 (0–0)	2.9 (0–16.2)	0 (0–7.9)	2.9 (0–11.8)	0 (0–5.9)	0 (0–5.9)	<0.001
AUSCAN physical function score^c^	0 (0–0)	0 (0–11.1)	2.8 (0–11.1)	2.8 (0–13.9)	0 (0–13.9)	0 (0–9.2)	<0.001

Differences in mean were tested using Anova, differences in median were tested using Kruskal–Wallis *H* test, and differences in frequencies were tested using Pearson Chi-square test

*GER* Germany, *IT* Italy, *NL* the Netherlands, *ES* Spain, *SWE* Sweden, *UK* the United Kingdom, *WOMAC* Western Ontario and McMaster Universities OA Index, *AUSCAN* Australian/Canadian OA Hand Index

^a^Mean (SD); ^b^ percentage; ^c^ median (IQ range)

**Fig. 2:**
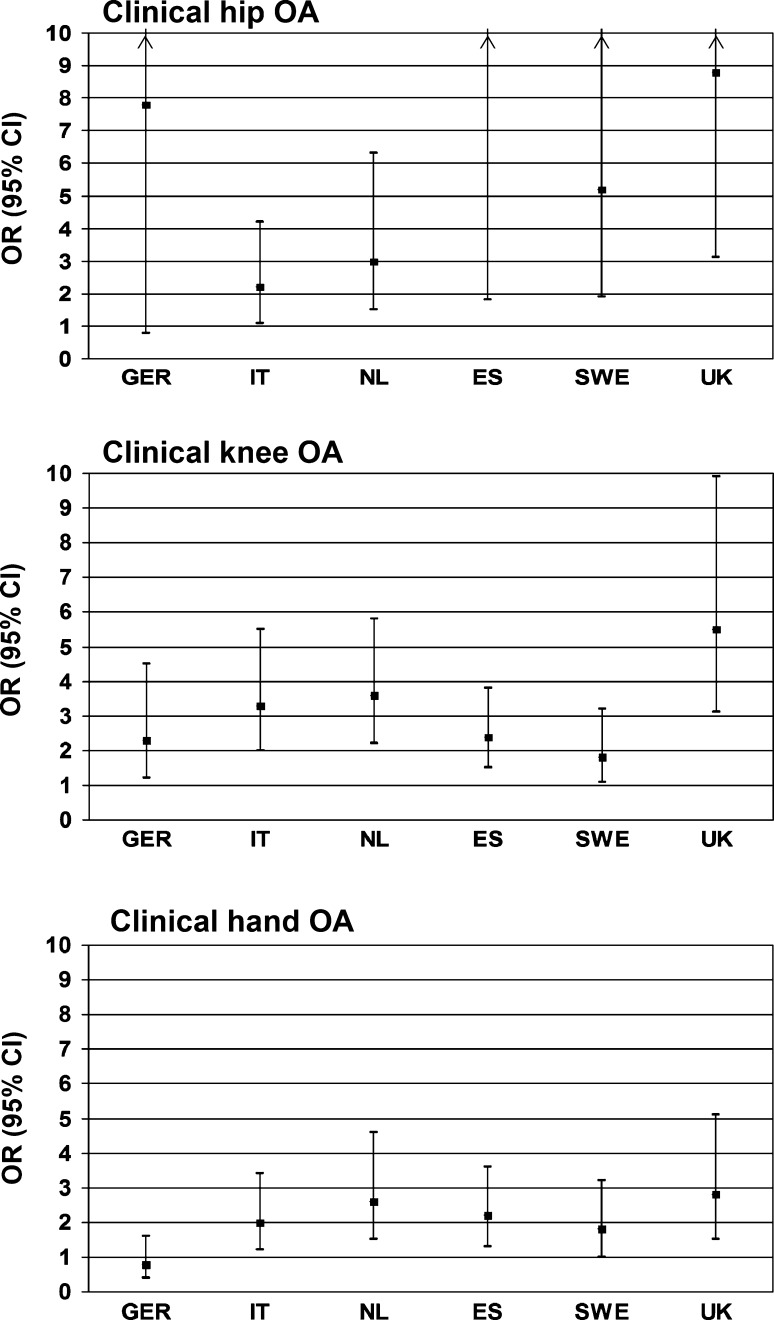
Cross-sectional association between clinical hip OA, clinical knee OA, clinical hand OA and fair-to-poor SRH stratified by country. *GER* Germany, *IT* Italy, *NL* the Netherlands, *ES* Spain, *SWE* Sweden, *UK* the United Kingdom. Logistic regression analyses were performed, and all analyses were adjusted for age, sex, educational level and number of chronic diseases. *OR* odds ratio, *95* *% CI* 95 % confidence interval

Country was a statistically significant effect modifier in the associations between clinical hip OA and SRH (*p* = 0.01 for IT, *p* = 0.09 for NL, *p* = 0.08 for SWE, analyzed as dummy variables using the UK as reference group); clinical knee OA and SRH (*p* = 0.07 for GER, *p* = 0.002 for SWE); and clinical hand OA and SRH (*p* = 0.03 for GER). Therefore, in Fig. 2, the associations between clinical OA and SRH are presented for each country separately. After adjustment for age, sex, educational level and number of chronic diseases, clinical OA of the hip, knee and hand were significantly associated with fair-to-poor SRH in all countries, except for GER, in which only knee OA was significantly associated with fair-to-poor SRH. However, all results (including hip OA in GER) pointed in the same direction. In Fig. 2, it can be seen that clinical knee OA and hand OA followed the same pattern in the six countries with the strongest associations observed in the UK and the weakest associations observed in SWE and GER. In general, the associations were weaker for hand OA as compared with knee OA. The pattern seems different for clinical hip OA. However, the confidence intervals were wide for clinical hip OA.

In Table 4, the mediation analyses are presented. As in Fig. 2, clinical OA at all sites was significantly associated with fair-to-poor SRH in all countries (i.e., the total effect), except for GER. Furthermore, all associations were less strong after adjustment for physical function (i.e., the direct effect). In some cases, the direct effect was still statistically significant, suggesting partial mediation. The indirect effect was statistically significant in most countries and at most sites, indicating that the association between clinical OA and fair-to-poor SRH was (partly) mediated by physical function.

**Table 4:** Physical function as a mediator in the association between clinical OA and fair-to-poor SRH: standardized total, direct and indirect effects (and 95 % CI)

	Beta (95 % CI) for hip OA^a^	Beta (95 % CI) for knee OA^a^	Beta (95 % CI) for hand OA^a^
GER			
Total effect	0.11 (−0.01 to 0.24)	0.15 (0.03 to 0.27)*	−0.03 (−0.16 to 0.11)
Direct effect	0.10 (−0.03 to 0.23)	0.13 (−0.002 to 0.25)	−0.01 (−0.16 to 0.13)
Indirect effect	^b^	0.03 (−0.04 to 0.10)	−0.02 (−0.14 to 0.05)
IT			
Total effect	0.14 (0.01 to 0.26)*	0.28 (0.16 to 0.40)*	0.13 (0.01 to 0.25)*
Direct effect	0.02 (−0.12 to 0.16)	0.17 (0.02 to 0.32)*	0.05 (−0.09 to 0.19)
Indirect effect	0.15 (0.06 to 0.31)*	0.11 (0.01 to 0.22)*	0.09 (0.02 to 0.16)*
NL			
Total effect	0.17 (0.06 to 0.28)*	0.26 (0.15 to 0.36)*	0.19 (0.08 to 0.30)*
Direct effect	0.06 (−0.05 to 0.18)	0.02 (−0.12 to 0.16)	0.13 (0.02 to 0.25)*
Indirect effect	0.14 (0.09 to 0.32)*	0.22 (0.15 to 0.32)*	0.06 (0.02 to 0.12)*
ES			
Total effect	0.28 (0.05 to 0.51)*	0.17 (0.06 to 0.29)*	0.16 (0.04 to 0.27)*
Direct effect	0.18 (−0.04 to 0.40)	0.01 (−0.13 to 0.14)	0.03 (−0.09 to 0.16)
Indirect effect	^c^	0.17 (0.10 to 0.25)*	0.14 (0.07 to 0.22)*
SWE			
Total effect	0.21 (0.10 to 0.33)*	0.14 (0.02 to 0.26)*	0.14 (0.01 to 0.26)*
Direct effect	0.13 (0.02 to 0.24)*	−0.01 (−0.16 to 0.14)	0.08 (−0.07 to 0.22)
Indirect effect	0.09 (0.05 to 0.24)*	0.14 (0.06 to 0.23)*	0.07 (−0.02 to 0.16)
UK			
Total effect	0.25 (0.13 to 0.37)*	0.32 (0.21 to 0.43)*	0.22 (0.10 to 0.33)*
Direct effect	0.20 (0.07 to 0.33)*	0.10 (−0.04 to 0.24)	0.06 (−0.07 to 0.19)
Indirect effect	0.04 (−0.02 to 0.11)	0.20 (0.12 to 0.29)*	0.16 (0.09 to 0.24)*

*OA* osteoarthritis, *GER* Germany, *IT* Italy, *NL* the Netherlands, *ES* Spain, *SWE* Sweden, *UK* the United Kingdom

Total effect: effect of OA on fair-to-poor SRH; Direct effect: effect of OA on fair-to-poor SRH after adjustment for physical function; Indirect effect: the multiplication of the effect of OA on physical function and the effect of physical function on fair-to-poor SRH after adjustment for OA

* Statistically significant at* p* < 0.05

^a^Standardized regression coefficient (and 95 % confidence interval) after adjustment for confounding variables

^b^Because of the very low prevalence of hip OA in GER and the high percentage of persons scoring “no difficulties” on the WOMAC physical function subscale, the indirect effect could not be calculated

^c^The indirect effect could not be calculated as there were no people having clinical hip OA in combination with a WOMAC score in the lowest three quartiles in Spain

## Discussion

In our study, clinical OA of the hip, knee and hand was associated with fair-to-poor SRH in the general population in five out of six European countries. Although the strength of the observed associations differed between countries, all pointed in the same direction. In most countries and at most sites, the association between clinical OA and fair-to-poor SRH was (partly) mediated by physical function.

To our knowledge, only few studies examined the association between OA and SRH in the general population [12–18]. In the studies using a self-reported medical provider diagnosis of OA [14, 15] or a self-reported diagnosis of arthritis (or rheumatism) [12, 16–18], OA was related to worse SRH, which is supported by the results of our study. In a study examining hip pain and knee pain in relation to SRH, only an independent association between hip pain and SRH was observed [13]. The above-mentioned studies were examined in one country only and only one study was performed in Europe [13].

In the current study, the prevalence of fair-to-poor SRH was highest in IT and ES. Similar results were observed in two earlier studies [19, 20], although in the first study, GER had similar levels of SRH as compared to IT and ES [19]. The higher prevalence of fair-to-poor SRH in IT and ES in our study may be explained in several ways. Firstly, by differences in population structure regarding the individual characteristics affecting SRH. In a study comparing SRH in IT and France, these differences in population structure, i.e. socio-demographic characteristics, diseases and disabilities, lifestyle, and others, were a more important explanation than country-specific relationships between these characteristics and SRH [37]. Also in the Comparison of Longitudinal European Studies on Aging (CLESA) study, homogenous associations between most indicators of medical and functional health and SRH were observed in the included countries [20]. Secondly, in a study published in 2007, it was shown that cross-national differences in self-reported health can partly be explained by differences in ‘true’ health (as measured by the prevalence of chronic conditions and objective health measures) and partly by cross-cultural differences in response style [38]. However, although the prevalence of fair-to-poor SRH was higher in Southern countries in our study, it is important to note that the observed associations between clinical OA of the hip, knee or hand and SRH were not stronger in Southern countries than in Northern and Western countries.

The associations between clinical OA of the knee and hand follow the same pattern in the different countries with the strongest associations observed in the UK and the weakest associations observed in SWE and GER (Fig. 2). This may partly be explained by differences in healthcare systems. In a study from 2001, the UK and SWE both were characterized by a medium level of total health expenditure, a high share of public health funding, moderate private out-of-pocket funding and highly regulated access to doctors; while Germany was characterized by a high level of total health expenditure, a high share of public funding, moderate private out-of-pocket funding and high freedom of choice for patients [39]. The lower impact of clinical OA on SRH in GER as compared with the UK and SWE might be explained by the higher level of total health expenditure and the higher freedom of choice for patients in GER. The latter might also lead to easier access to medical doctors. The difference between the UK and SWE may be explained by the fact that the total health expenditure is higher in SWE than in the UK [39]. Further research might address the role of differences in healthcare systems in the observed country differences.

Although hip OA was significantly associated with fair-to-poor SRH in five out of six countries, the confidence intervals of the ORs were very wide (Fig. 2), which may be explained by the relatively low incidence of clinical hip OA in the general population. Larger studies in the general population are needed to draw a final conclusion about the strength of the associations between clinical hip OA and fair-to-poor SRH and about the pattern of the associations across countries.

In GER, clinical OA of the hip and hand were not significantly related to fair-to-poor SRH, although it should be noted that the results for hip OA were in the same direction as compared with the other countries. An important symptom of clinical OA is pain. In contrast to the other countries, in GER, an index question was used to assess whether participants had pain at the hip, knee or hand, before the specific WOMAC/AUSCAN pain questions were asked [6]. In case no pain was reported on this index question, the WOMAC/AUSCAN pain questions were automatically scored as “no pain.” This may have led to a lower estimated prevalence of clinical OA in Germany and to an underestimation of the observed associations.

In addition to physical function, pain, may also (partly) mediate the association with fair-to-poor SRH. However, because pain is part of our OA definition, it was strongly correlated with OA and we decided not to examine whether the observed associations were mediated by pain. In an earlier study on self-reported arthritis and self-rated health, pain and activity limitations fully accounted for the associations between arthritis onset and worsening SRH [12].

Our study has several strengths. First, we studied the association between clinical OA and fair-to-poor SRH in population-based samples, representative for the general older population, in six different European countries. We used identical measurements in all countries, including a clinical measurement of OA, which makes this the first European study in which the impact of clinical OA on fair-to-poor SRH can be compared between countries. Limitations include the relatively low power for clinical hip OA in our study due to the low incidence of clinical hip OA in the general population. In addition, as EPOSA was set up as a side study in existing cohort studies (except for the IT sample), there may have been differences in recruitment procedures (see design paper for more details [6]). Finally, people who were excluded due to missing data were older and had lower educational level. As a result, the observed associations may be underestimated.

In conclusion, while SRH varied across the European countries, clinical OA of the hip and hand were significantly associated with fair-to-poor SRH in the general population in five European countries; and clinical knee OA was significantly associated with fair-to-poor SRH in six European countries. Most of the observed associations were (partly) mediated by poor physical function, indicating that deteriorating physical function in patients with OA should be a point of attention in patient care. Further research might address the role of differences in healthcare systems in the observed country differences.
